# Supplementary material for: Identification of photoperiod-induced specific miRNAs in the adrenal glands of Sunite sheep (Ovis aries)
Source: Front Vet Sci. 2022 Jul 22;9:888207. doi: 10.3389/fvets.2022.888207 (PMC9354845; doi:10.3389/fvets.2022.888207)
Supplement: Supplementary file 1 [file Data_Sheet_1.ZIP › Supplementary file/miRDeep2 file.zip!/miRDeep2 file/Novel_Structure/SPLP42A2/Novel_344.pdf]

Provisional ID	:	NC_019482.2_40623
Score total	:	2
Score for star read(s)	:	-1.3
Score for read counts	:	-0.3
Score for mfe	:	2.1
Score for randfold	:	1.6
Score for cons. seed	:	
Total read count	:	11
Mature read count	:	11
Loop read count	:	0
Star read count	:	0

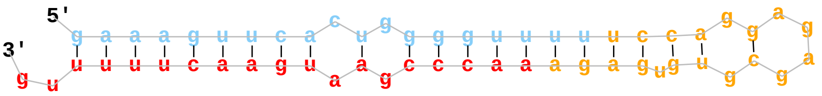

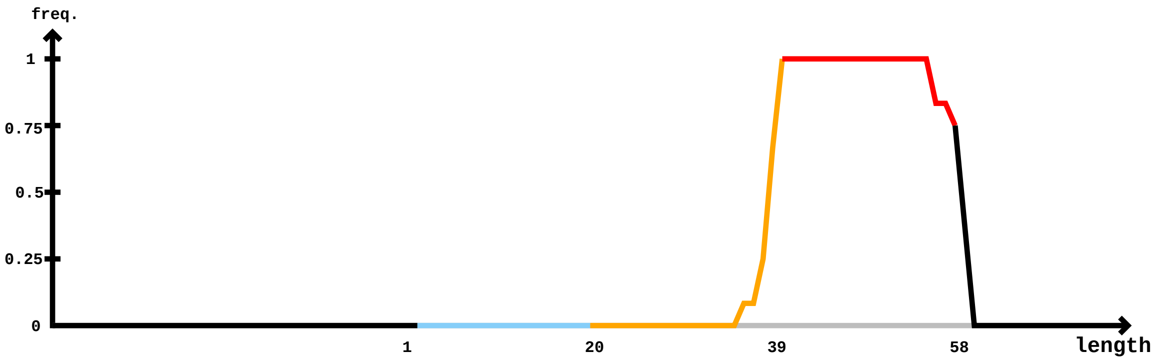

Star Mature

```
5'- uggccacaaucaauuuugguauauacuggauuggcugaaaguucacugggguuuuuccaggagagcgugugagaaacccgaaugaacuuuuugaccaaccaguauauaua  -3'   exp
    ......(((......)))(((((((((((.(((((.(((((((((.(.(((((((((((.(....).)).))))))))).).))))))))).).))))))))))))))).    reads    mm    sample
    ......................................................................gagaaacccgaaugaacuuu....................    1    0    seq
    ........................................................................gaaaccUgaaugaacuuuuug.................    2    1    seq
    .........................................................................aaacccgaaugaacuuu....................    1    0    seq
    .........................................................................aaacccgaaugaacuuuuuC.................    1    1    seq
    .........................................................................aaacccgaaugaaAuuuuug.................    1    1    seq
    .........................................................................aaaccAgaaugaacuuuuug.................    1    1    seq
    .........................................................................aaacccAaaugaacuuuuuga................    1    1    seq
    ..........................................................................aacccgaaugaacuuuuu..................    1    0    seq
    ..........................................................................aacccgaaugaacuuuuuC.................    1    1    seq
    ..........................................................................aacccgaaugaacuuuuug.................    2    0    seq
```
